# Management of Locally Advanced Renal Cell Carcinoma with Invasion of the Duodenum

**DOI:** 10.1155/2013/596362

**Published:** 2013-04-03

**Authors:** Andrew T. Schlussel, Aaron B. Fowler, Herbert K. Chinn, Linda L. Wong

**Affiliations:** ^1^Department of General Surgery, Tripler Army Medical Center, 1 Jarrett White Road, Honolulu, HI 96859, USA; ^2^University of Utah, School of Medicine, 30 North 1900 East, Salt Lake City, UT 84132, USA; ^3^Department of Urology, Queens Medical Center, 1329 Lusitana Street, Suite 108, Honolulu, HI 96813, USA; ^4^Department of Surgery, University of Hawaii School of Medicine, 550 South Beretania Street, Suite 403, Honolulu, HI 96813, USA

## Abstract

Renal cell carcinoma (RCC) is rare but aggressive, with greater than 20% of patients presenting with stage III or IV, disease. Surgical resection of the primary tumor regardless of stage is the treatment of choice, and en bloc resection of involved organs provides the only potential chance for cure. This case report describes a patient with metastatic right-sided RCC with invasion of the inferior vena cava and duodenum managed by en block resection and pancreaticoduodenectomy. This report will review the workup and treatment of locally advanced RCC, as well as the role of cytoreductive nephrectomy in the setting of metastatic disease.

## 1. Introduction

Renal cell carcinoma (RCC) is a relatively rare cancer that comprises approximately 2% of newly diagnosed visceral cancers in the United States. Tobacco and obesity are the most significant risk factors and are present in 20% and 30% of renal cell carcinoma, respectively [1]. RCC most often develops in the sixth and seventh decades of life with a male-to-female ratio of 2 : 1. It is estimated that in 2012 there will be 64,770 new cases of renal cancer and 13,570 deaths from this malignancy [2].

The most common site of invasion for right-sided renal cell carcinoma is the inferior vena cava (IVC) causing thrombus formation. Previous studies have demonstrated that surgical intervention with enbloc removal of the tumor thrombus in these cases improves overall survival [3]. To date, the mainstay of therapy for RCC invading the IVC involves a radical nephrectomy, cavotomy, and thrombus extraction followed by immunotherapy [4]. Haferkamp et al. demonstrated that surgical resection alone increases survival, but when combined with adjuvant immunotherapy these rates were dramatically increased [4].

Although uncommon, metastatic renal cell carcinoma to the duodenum has been described; however, direct invasion from the kidney into the duodenum has not been reported [5]. Furthermore, there have been no reports of renal cell carcinoma invading both the duodenum and IVC. We present a case of a patient with RCC of the right kidney with invasion of the inferior vena cava and duodenum as well as subsequent treatment.

## 2. Case Report

**Figure 1:**
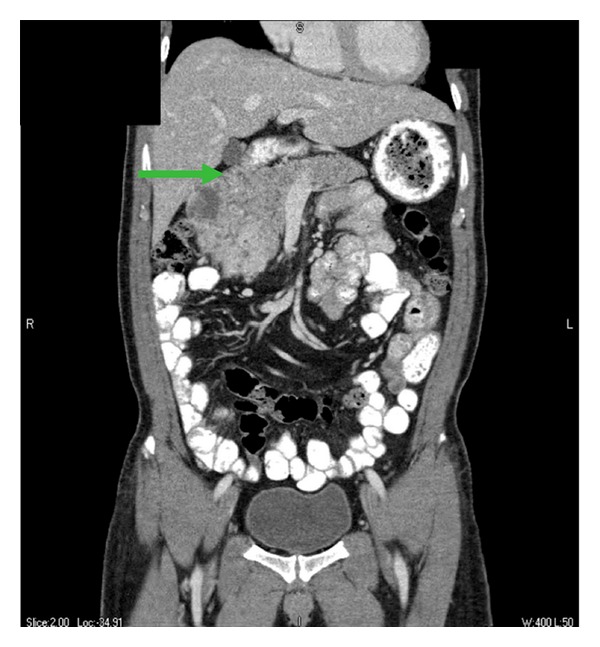
Displacement of duodenum by right kidney mass. Arrow indicates duodenum.

**Figure 2:**
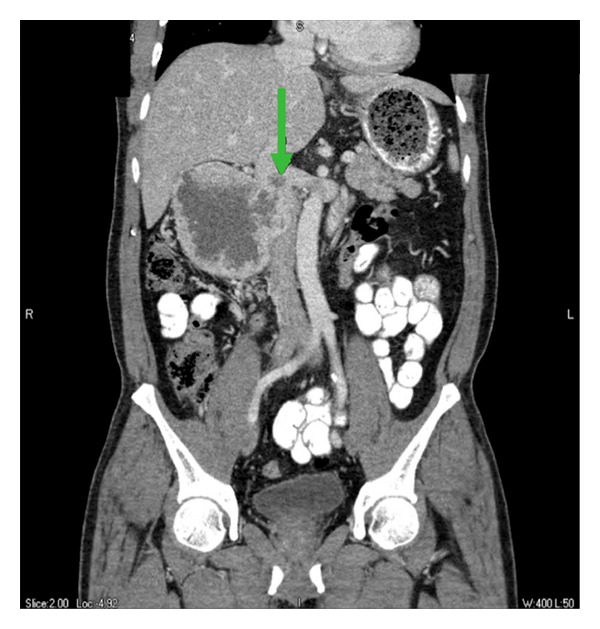
Extension of right kidney mass into the lumen of the inferior vena cava. Arrow annotates the inferior vena cava and tumor thrombus.

This is a case of a 53-year-old Filipino male with a past medical history significant for hypertension and diabetes mellitus, who presented with symptoms of melena, fatigue, and lightheadedness. He denied abdominal pain, nausea, vomiting, fevers, chills, anorexia, or weight loss. He otherwise had an excellent performance status and no family history of cancer. His physical exam was normal without any dominant palpable abdominal mass or leg edema to suggest venous congestion or thrombus. The laboratory workup was significant for a hemoglobin of 6.6 gm/dL for which he received a transfusion of six units of packed red blood cells. He underwent an esophagogastroduodenoscopy that showed a bleeding mass involving the second portion of the duodenum. Hemostasis was achieved and a biopsy was performed that was consistent with renal cell carcinoma. Subsequently, a computed tomography (CT) scan was performed and showed a large mass arising off the anterior cortex of the lower pole of the right kidney with brisk arterial and peripheral enhancement consistent with renal cell carcinoma. The dimensions of this mass were estimated to be approximately 10.1 cm by 8.0 cm by 10.0 cm, protruding into the lumen of the duodenum and displacing it medially (Figure 1). Coronal reformatted images obtained of the portal venous phase demonstrated extension of the mass into the lumen of the inferior vena cava (Figure 2). It appeared that the majority of the infrarenal IVC was displaced and compressed medially. Magnetic resonance imaging (MRI) confirmed the findings on CT scan, and a positron emission tomography (PET) scan was also performed, which demonstrated a large hypermetabolic mass involving the right kidney without evidence of regional metastasis. CT-guided imaging of the patient's chest revealed a cluster of pulmonary nodules and irregular opacities in the left upper lobe and right upper lobe that were hypermetabolic on PET scan and consistent with metastatic disease. The patient was evaluated in a multidisciplinary tumor board, and based on the current literature it was the consensus that surgical resection be attempted with the plan for adjuvant immunotherapy postoperatively [6–8].

**Figure 3:**
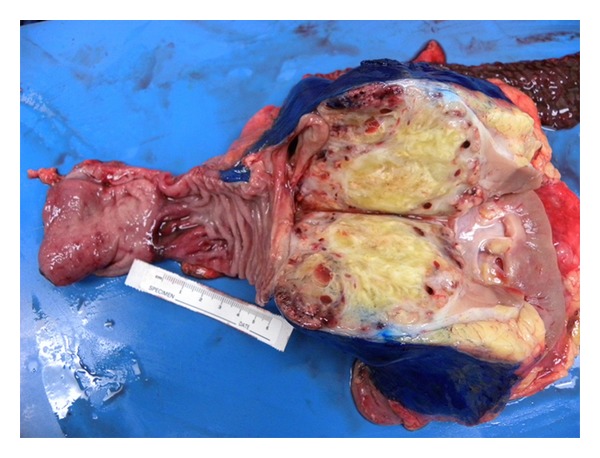
Right renal mass invading the duodenum. Ruler demonstrates length of duodenal invasion.

**Figure 4:**
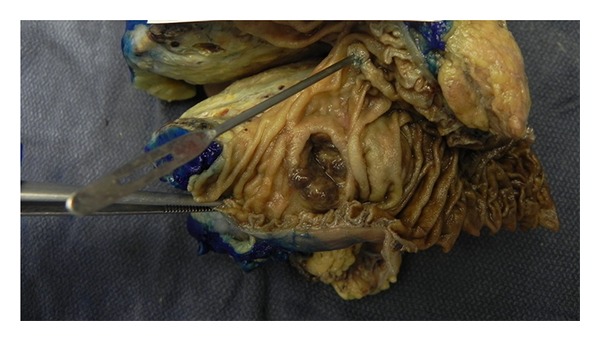
Luminal view of duodenum with invasion of the second portion.

Exploratory laparotomy revealed a 12 cm by 15 cm tumor in the right kidney with extension into the inferior vena cava as well as a second 3 cm by 4 cm irregular mass partially adherent to the right renal vein and obstructing the vena cava. There was no evidence of intraabdominal metastases outside these areas. There was extension of the tumor into the second portion of the duodenum but not obstructing the bile duct or invading the portal vein (Figures 3 and 4). Therefore, a right radical nephrectomy was performed with en bloc pancreaticoduodenectomy and resection of the inferior vena cava tumor thrombus with the use of a modified venovenous bypass. A 17-French catheter was inserted in the left femoral vein with blood return at a flow rate of 1.5 L/min to three large bore catheters placed in the bilateral internal jugular veins. The IVC was temporarily clamped for approximately 15 minutes in order to remove the intravascular tumor. At the completion of the procedure, the IVC appeared to be slightly narrowed at about 1.8 cm, but this was distal to the insertion of the left renal vein, and the flow appeared to be adequate. Histological evaluation demonstrated a pT4pN0M1 stage IV, grade 3, clear cell renal carcinoma with direct involvement of the duodenum. The patient tolerated the procedure well without any major intraoperative complications. The total operative time was approximately six hours and his blood loss was five liters. He required a transfusion of ten units of packed red blood cells, eight units of fresh frozen plasma, and one unit of platelets. He had an uncomplicated postoperative course. His creatinine initially increased to a maximum of 1.7 mg/dL from a baseline of 0.9 mg/dL, but at the time of discharge the value had decreased and stabilized at 1.1 mg/dL. His diet was advanced slowly and he underwent gastrografin swallowing study on postoperative day seven, which demonstrated no evidence of anastomotic leak, stenosis, or delayed gastric emptying. He was discharged home on postoperative day ten tolerating a soft diet and in stable condition. The patient has recovered and is now three months after surgery without any complications. He is expected to undergo adjuvant therapy with interleukin-2 in the near future.

## 3. Discussion

Locally advanced RCC occurs in about 2% of the population, and at initial presentation 26.7% are diagnosed with stage II or III disease, with up to 22.7% having metastatic spread [9]. En bloc resection of all involved organs is the only potential curable operation [10]. Typically patients at this stage of the disease will present with pain due to invasion of the posterior abdominal wall, nerve roots, and paraspinous muscles. It is uncommon for these tumors to invade adjacent organs to include the liver, duodenum, and pancreas. Unfortunately such extensive disease portends a poor prognosis with reports of a 5% survival rate at 5 years after margin negative resection [10].

Being a technically challenging operation, a combined radical nephrectomy with pancreaticoduodenectomy and IVC resection is rarely performed, and the indications are not well defined [10]. Left-sided RCC with pancreatic invasion has also been described, where en bloc resection with distal pancreatectomy was the treatment of choice [11]. In one study, only 5 out of 180 patients, over 6 years, required a concomitant nephrectomy and pancreaticoduodenectomy. The procedure was performed for three retroperitoneal sarcomas, one locally advanced transitional-cell carcinoma, with only adherence to the duodenal wall not true invasion, and an ampullary cancer with a concurrent right renal cell tumor. All patients did well, with three (60%) reported to have complications related to the pancreaticoduodenal resection, a procedure alone that has a significantly high morbidity rate [12, 13].

Metastatic renal cell carcinoma to the duodenum has been reported and is rare [14]. Pancreatic metastases occur in 1.3–1.9% of patients based on autopsy results. This occurs via the hematogenous route and is classically seen in patients many years after resection of the primary tumor. More common sites for metastatic disease include the lungs, bone, liver, renal fossa, and brain, where the small intestine comprises only 1-2% of all metastases from any tumor. Surgical resection for metastatic RCC is associated with a 5-year survival rate of 29%–35%, and pancreaticoduodenectomy has been described as a method to clear disease [14]. Attempt at complete resection maintains the best outcome; however, arteriography with embolization of the gastroduodenal artery and duodenal wedge resection has also been described for palliation in the event of a massive gastrointestinal bleed due to duodenal metastasis [5, 14–16].

The workup and management of locally advanced RCC with direct invasion of the duodenum are complex and can only be extrapolated from that of metastatic RCC and the knowledge of other locally invasive cancers requiring pancreaticoduodenectomy [10, 17]. Although these renal tumors demonstrate an aggressive biology, it has been shown in patients with pancreatic metastasis that there is a more favorable prognosis than with resection for a primary pancreatic carcinoma [13]. The patient described here is complicated further by the involvement of the inferior vena cava, which occurs in 4%–10% of patients with renal cell carcinoma. This was once thought to have a very poor prognosis; however, early surgical intervention has been shown to have a 5-year survival rate of 45%–69%, but when confined to the kidney alone [10].

Cytoreductive nephrectomy, an upfront aggressive surgical resection of the renal primary tumor in the face of known metastatic disease, remains the standard treatment of stage IV RCC [6–8]. However, such a radical resection should only be attempted in patients with good performance status, those with minimal comorbidities, an overall low surgical risk, no hepatic, brain or skeletal metastasis, and when 75% if the tumor bulk can be excised [9]. Flanigan and colleagues were able to demonstrate an overall survival benefit of 13.3 months versus 7.8 months with the use of cytoreductive nephrectomy and interferon (IFN) compared to IFN alone in metastatic RCC [7]. Interleukin-2 has also demonstrated some benefit in the adjuvant setting, with complete response rates occurring between 5 and 9% of the time. These therapies, although effective, are associated with significant toxicity [9]. Currently there is no other prospective analysis proving the efficacy of adjuvant therapy. Phase III trials are currently studying the safety of sunitinib in combination with cytoreductive nephrectomy [9]. There is also no conclusive evidence that recommends the use of neoadjuvant therapy for locally advanced RCC [18]. There are some reports that therapy may decrease or downstage the caval tumor thrombus, but partial tumor response is low and complete response is rare. This remains a controversial subject, and prospective studies are ongoing evaluating the potential benefit, timing, and safety of neoadjuvant therapies [8, 9].

In conclusion, this patient was successfully managed based on the current medical literature of locally advanced RCC with IVC involvement. The patient had an extensive preoperative workup with a high-quality CT scan and MRI, which provided appropriate preparation for the surgical team. He received an aggressive and an oncologically sound operation, providing the best potential chance for survival.
